# Effect of icosapent ethyl on susceptibility to ventricular arrhythmias in postinfarcted rat hearts: Role of GPR120‐mediated connexin43 phosphorylation

**DOI:** 10.1111/jcmm.15575

**Published:** 2020-07-08

**Authors:** Wei‐Ting Chen, Syue‐yi Chen, De‐Wei Wu, Cheng‐Che Lee, Tsung‐Ming Lee

**Affiliations:** ^1^ Cardiovascular Institute An Nan Hospital China Medical University Tainan Taiwan; ^2^ Tainan First Senior High School Tainan Taiwan; ^3^ Municipal HouJia Junior High School Tainan Taiwan; ^4^ Department of Medicine China Medical University Taichung Taiwan

**Keywords:** arrhythmias, connexin43, G protein‐coupled receptor 120, myocardial infarction, ω‐3 polyunsaturated fatty acids

## Abstract

The ω‐3 fatty acids exert as an antioxidant via the G protein‐coupled receptor 120 (GPR120). Icosapent ethyl, a purified eicosapentaenoic acid, showed a marked reduction in sudden cardiac death. Connexin43 is sensitive to redox status. We assessed whether icosapent ethyl attenuates fatal arrhythmias after myocardial infarction, a status of high oxidative stress, through increased connexin43 expression and whether the GPR120 signalling is involved in the protection. Male Wistar rats after ligating coronary artery were assigned to either vehicle or icosapent ethyl for 4 weeks. The postinfarction period was associated with increased oxidative‐nitrosative stress. In concert, myocardial connexin43 levels revealed a significant decrease in vehicle‐treated infarcted rats compared with sham. These changes of oxidative‐nitrosative stress and connexin43 levels were blunted after icosapent ethyl administration. Provocative arrhythmias in the infarcted rats treated with icosapent ethyl were significantly improved than vehicle. Icosapent ethyl significantly increased GPR120 compared to vehicle after infarction. The effects of icosapent ethyl on superoxide and connexin43 were similar to GPR120 agonist GW9508. Besides, the effects of icosapent ethyl on oxidative‐nitrosative stress and connexin43 phosphorylation were abolished by administering AH‐7614, an inhibitor of GPR120. SIN‐1 abolished the Cx43 phosphorylation of icosapent ethyl without affecting GPR120 levels. Taken together, chronic use of icosapent ethyl after infarction is associated with up‐regulation of connexin43 phosphorylation through a GPR120‐dependent antioxidant pathway and thus plays a beneficial effect on arrhythmogenic response to programmed electrical stimulation.

## 1 INTRODUCTION

ω‐3 polyunsaturated fatty acids (PUFAs) have been identified as vital nutrients to provide energy and play important roles in signalling transduction that might be able to offer benefits in a wide range of diseases. Epidemiological studies have shown that the mortality following myocardial infarction (MI) was reduced by 29% in the group advised to consume oily fishes compared with the non‐fish groups during the follow‐up of 2 years.^1^ This reduced mortality was not associated with reduced ischaemic cardiovascular events or levels of total cholesterol. Antiarrhythmic effects of ω‐3 PUFAs may play a role in reducing mortality including sudden cardiac death after MI.^2^ Increased content of ω‐3 PUFAs eicosapentaenoic acid (EPA) and docosapentaenoic acid (DHA) in red blood cells has been associated with a markedly reduced risk for sudden cardiac death.^3^ A recent guideline suggested that benefits of ω‐3 PUFAs may be obtained through seafood consumption as with dietary supplementation.^4^ However, some studies have not reported a cardioprotective effect with ω‐3 PUFA treatment, and animal model studies have even reported a proarrhythmic effect during acute regional myocardial ischaemia.^5^ Raitt et al^6^ reported that recurrent ventricular fibrillation or ventricular tachycardia occurred more often in the ω‐3 PUFA group in patients with implantable defibrillators and may be proarrhythmic. Thus, the effect of ω‐3 PUFAs on ventricular arrhythmia remains undetermined and more studies are required.

Basic research has shown that enrichment of myocardial membranes with ω‐3 PUFAs reduces arrhythmic vulnerability.^7^ Although the long‐chain ω‐3 PUFAs have been shown to incorporate into membrane phospholipids, resulting in changes in gene expression profiles,^8^ EPA has been shown to inability of accumulation in the membrane of cardiac myocytes and nonmyocytes (fibroblasts) at effective dose,^9^ suggesting an alternate mechanism for EPA‐mediated effects. EPA has been shown to attenuate superoxide production and prevent vascular calcification in *klotho* mutant mice.^10^ Furthermore, Resolvin E1, a downstream mediator derived from EPA, can suppress reactive oxygen species (ROS) production at low concentrations.^11^ Thus, we hypothesized that EPA has beneficial effects on postinfarction arrhythmias by attenuating ROS production.

Connexin43 (Cx43) is the major structural protein of ventricular gap junctions, and a significant decrease in Cx43 causes sudden arrhythmic death.^12^ Reduction of gap junctions in cardiac injury is associated with increased arrhythmogenicity.^13^ Increased Cx43 levels by gene transfer ameliorate arrhythmia susceptibility in the border zone after MI.^14^ Previous studies have shown that ROS production reduces Cx43 at intercalated discs through competition with activated c‐Src and increases ventricular arrhythmias in a mouse model of cardiac renin‐angiotensin system activation.^15^

The G protein‐coupled receptor 120 (GPR120) is an ω‐3 fatty acid receptor which is up‐regulated when EPA is administered at micromolar concentrations.^16^ GPR120 was highly expressed in the myocardium. The high level of GPR120 expression in myocardium indicates that GPR120 might play an important role in cardiomyocytes, such as antioxidation. Very recently, GPR120 activation has been shown to regulate redox status via GPR120‐nuclear factor E2‐related factor 2 (Nrf2) crosstalk mechanism.^17^ *GPR120* gene knockdown by siRNA cancelled effects of EPA on ROS production,^10^ implying that GPR120 mediated the inhibitory effects of EPA on ROS production. Intake of ω‐3 PUFAs was associated with up‐regulation of Cx43 in diabetic^18^ and hypertensive^19^ animals. However, the role of ω‐3 PUFAs in modifying Cx43 after MI has not previously been described. Therefore, in this study, we evaluated whether the administration of EPA could attenuate arrhythmias postinfarction through GPR120 and by increasing the expression of Cx43. The aims of this study were to (a) investigate whether the chronic administration of icosapent ethyl (IPE), a highly purified synthetic derivative of EPA, could attenuate arrhythmias by enhancing the expression of Cx43; and (b) assess the role of GPR120 in regulating the expression of Cx43 in a rat MI model using an agonist and antagonist of GPR120.

## 2 METHODS

The animal experiment was approved and conducted in accordance with the local ethical review committee on animal care of the China Medical University and conformed with the *Guide for the Care and Use of Laboratory Animals* published by the US National Institutes of Health (NIH Publication No. 85‐23, revised 1996).

### 2.1 Animals

#### 2.1.1 Experiment 1: in vivo

The anterior descending arteries of male Wistar rats (200‐250 g) were ligated as previously reported,^20^ causing left ventricular (LV) free wall infarction. Rats were randomly assigned into either vehicle (saline) group or IPE (VASCEPA^®^, 0.3 g/kg per day; Amarin Pharma Inc) by gastric intubation in a final volume of 1.0 mL once a day. Rats were fed with IPE at a dose equivalent to the maximum clinical dose of 4 g/d based on body surface area comparison. Our intention was to achieve effects closer to levels observed in patients treated with prescription EPA supplementation in clinical trials.^21^ Tap water and standard diet were provided ad libitum.

Treatment with the drug was started 24 hours after infarction by daily oral gavage, as maximum benefits of the treatment would be achieved at this time point.^22^ The study was conducted over a 4‐week period, as most (70%‐80%) of the myocardial remodelling process in rats occurs within 3 weeks.^23^ Sham‐operated rats were used as controls to rule out the possible direct effect of the drug itself on the phosphorylation of Cx43. To eliminate the pharmacological actions of the drug, treatment was stopped approximately 24 hours prior to the end of the experiment.

#### 2.1.2 Experiment 2: ex vivo

To elucidate the potential role of GPR120 in mediating ROS, we performed ex vivo experiments. Four weeks after induction of MI by coronary ligation, infarcted rat hearts were isolated and subjected to no treatment (vehicle), IPE (50 µmol/L), GW9508 (a GPR120 agonist, 5 µmol/L, 4‐{[(3‐phenoxyphenyl)methyl] amino}benzenepropanoic acid, Tocris bioscience), IPE + AH‐7614 (a GPR120 antagonist, 7 µmol/L, 4‐methyl‐*N*‐9*H*‐xanthen‐9‐yl‐benzenesulfonamide, Sigma‐Aldrich). The concentration of IPE (50 µmol/L) used in this study was based on free fatty acid levels in patients who were taking 5 g/d fish oil, comparable to plasma levels found in populations consuming high dietary fish intake.^24^ In addition, to avoid the uncertainty in results with the in vitro use of an EPA membrane, we used GW9508, a synthetic GPR120 agonist. GW9508 is a small molecule composed of benzyl ring structures, and as such it is unlikely to be acylated into membrane lipids, thereby allowing the discrimination between receptor activation and membrane incorporation. AH‐7614 has been shown to inhibit GPR120‐mediated signals induced by a range of distinct fatty acids and synthetic GPR120 agonist ligands.^25^ AH‐7614 is a simple xanthene‐containing chemical, and this molecule does not block agonist effects at GPR40.^26^ Each heart was perfused with a noncirculating modified Tyrode's solution as previously described.^27^ Drugs were infused for 1 hour, which was effective in attenuating ROS production.^28^ At the end of the study, all hearts (n = 5 per group) were used for ROS and Cx43 analysis in the remote zone (>2 mm outside the infarct).

#### 2.1.3 Experiment 3: ex vivo

Although results of the above study showed that IPE modulated ROS and Cx43 (see Section 3), the relationship between the two remained unclear. We employed 3‐morpholinosydnonimine (SIN‐1, a peroxynitrite generator) in an ex vivo experiment. Four weeks after MI had been induced by coronary artery ligation, the infarcted rat hearts were isolated and received treatment with IPE (50 µmol/L), IPE + SIN‐1 (37 µmol/L) or no treatment (vehicle). The dose of SIN‐1 was chosen as previous studies.^29^ The perfusion setting was the same as the Part 2. At the end of the study, all hearts (n = 5 each group) were used for measuring GPR120 and Cx43 levels.

### 2.2 In vivo electrophysiological studies

At the end of the study, the rats were intraperitoneally anaesthetized with Zoletil (20 mg/kg body weight) and xylazine (9 mg/kg). To assess the potential arrhythmogenic risk of reduced Cx43 protein, we performed in vivo programmed electrical stimulation after left thoracotomy and artificial respiration. Body temperature was maintained at 37°C with a thermostatically controlled heating lamp. Programmed electrical stimulation was performed with electrodes sewn to the epicardial surface of the right ventricular outflow tract. Pacing pulses were generated from a Bloom stimulator (Fischer Imaging Corporation). To induce ventricular arrhythmias, pacing was performed at a cycle length of 120 ms (S_1_) for eight beats, followed by one to three extrastimuli (S_2_, S_3_ and S_4_) at shorter coupling intervals. The end‐point of ventricular pacing was induction of ventricular tachyarrhythmia. Ventricular tachyarrhythmias including ventricular tachycardia and ventricular fibrillation were considered nonsustained when it lasted ≤15 beats and sustained when it lasted >15 beats. An arrhythmia scoring system was modified as previously described.^23^ 0, noninducible preparations; 1, nonsustained tachyarrhythmias induced with three extrastimuli; 2, sustained tachyarrhythmias induced with three extrastimuli; 3, nonsustained tachyarrhythmias induced with two extrastimuli; 4, sustained tachyarrhythmias induced with two extrastimuli; 5, nonsustained tachyarrhythmias induced with one extrastimulus; 6, sustained tachyarrhythmias induced with one extrastimulus; and 7, tachyarrhythmias induced during the eight paced beats. If the heart stopped before the pacing, the arrhythmia score assigned to that heart was 8. When multiple forms of arrhythmias occurred in one heart, the highest score was used. The experimental protocols were typically completed within 10 minutes.

### 2.3 Haemodynamics and infarct size measurements

At completion of the electrophysiological tests, the atria and the right ventricle were trimmed off, and the LV was rinsed in cold physiological saline, weighed and immediately frozen in liquid nitrogen after obtaining a coronal section of the LV for infarct size estimation. Each section was stained with haematoxylin and eosin, and trichrome. The infarction size was determined in sections taken from the equator of the LV. Infarct size was reported as an average of the proportions of LV endocardial and epicardial circumferences occupied by the infarct as previously described.^27^ With respect to clinical importance, only rats with large infarction (>30%) were selected for analysis. Haemodynamic parameters were measured (Appendix S1).

### 2.4 In situ detection of superoxide and nitrotyrosine

To measure myocardial superoxide and nitrotyrosine production, we used in situ dihydroethidium (DHE) and anti‐nitrotyrosine antibody. For a detailed method, please refer to the Appendix S1.

### 2.5 Real‐time RT‐PCR of GPR120 and Cx43

*GPR120* and *Cx43* mRNAs were quantified by real‐time quantitative reverse transcription‐polymerase chain reaction (RT‐PCR) using TaqMan system with *cyclophilin* as a loading control. Sequence of PCR primers are shown in the Appendix S1.

### 2.6 Western Blot analysis of GPR120, ser368‐phosphorylated Cx43 and total Cx43

For a detailed method, please refer to the Appendix S1.

### 2.7 Immunohistochemical studies of GPR120 and Cx43

In order to investigate the spatial distribution and quantification of GPR120 and Cx43, immunohistochemical analysis was conducted on LV muscles from the remote zone as Appendix S1.

### 2.8 Laboratory measurements

Blood samples from ascending aortas of the rats were collected at the end of the study, and serum was separated by centrifugation. To assure EPA levels more relevant to human populations, serum Resolvin E1 levels were measured by using a commercially available kit (MyBiosource, Inc) and following the manufacturer's instructions.

Myocardial production of superoxide in the remote zone was evaluated with lucigenin (5 µmol/L bis‐N‐methylacridinium nitrate, Sigma)‐enhanced chemiluminescence as reported in a previous study.^20^ The chemiluminescent signal was calculated by subtracting background activity and was expressed as counts/min/mg weight (cpm/mg).

Myocardial peroxynitrite formation was estimated by measuring the level of free nitrotyrosine (as a marker for peroxynitrite formation) by ELISA (Cayman Chemical) in myocardial homogenates.

### 2.9 Statistical analysis

Results were presented as mean ± SD. All statistical analyses were performed using SPSS software (SPSS, version 23.0). Differences among the groups of rats were tested by analysis of variance (ANOVA), with Bonferroni correction if there was a significant effect. Electrophysiological data (programmed electrical stimulation‐induced arrhythmia scores) were compared using the Kruskal–Wallis test followed by the Mann–Whitney test. A *P* value < .05 was considered to indicate a statistically significant difference.

## 3 RESULTS

### 3.1 Part 1: in vivo

**FIGURE 1:**
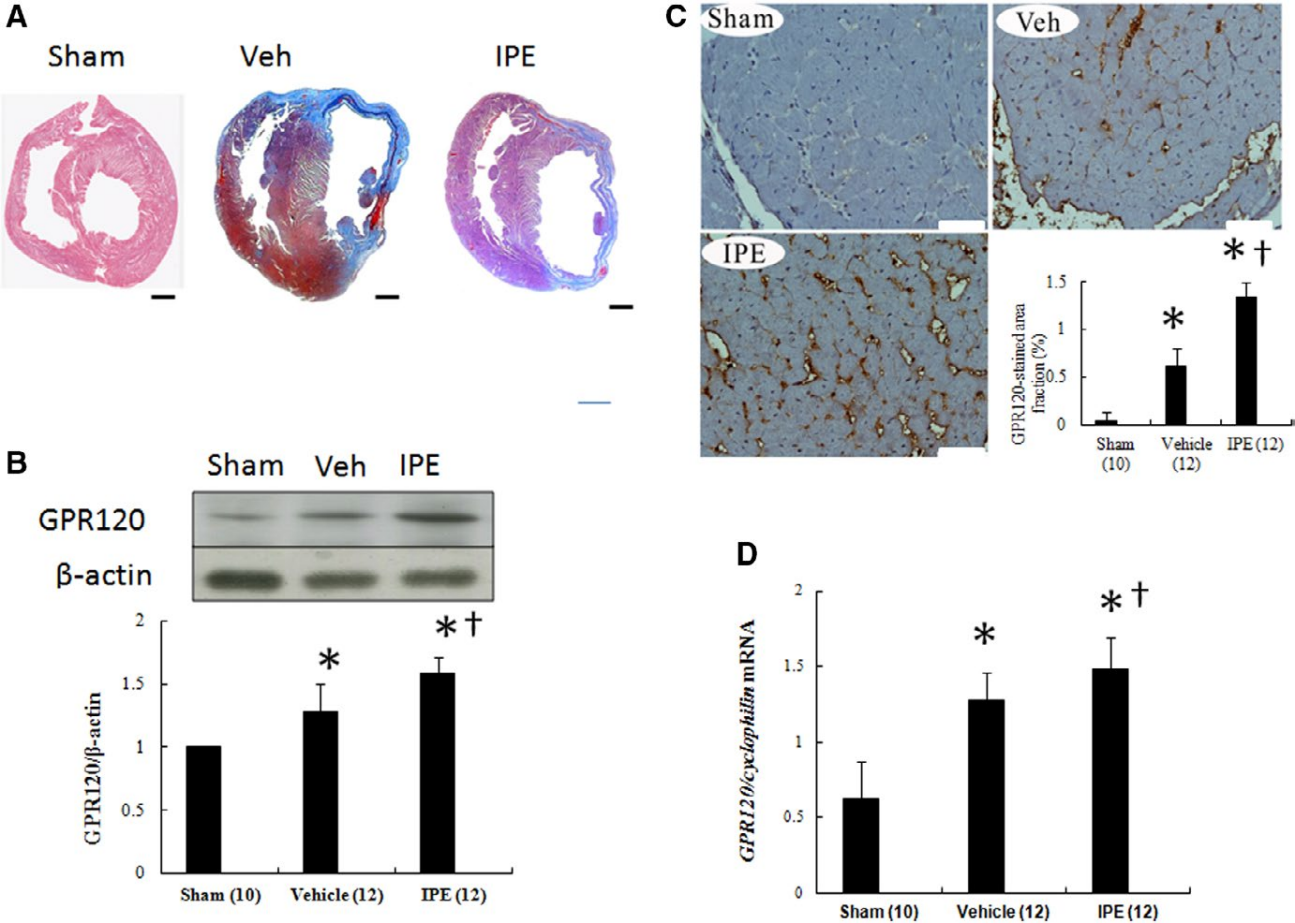
A, Representative Masson trichrome‐stained section at the end of the study. Bar = 2 mm. B, Western blot. When compared with vehicle‐treated infarcted rats, IPE‐treated infarcted rats had significantly higher GPR120 levels in the remote zone by quantitative analysis. Relative abundance was obtained by normalizing the density of GPR120 protein against that of β‐actin. C, Immunohistochemical staining of GPR120. GPR120 was observed to be localized at cell surface membrane. Bar = 50 μm. D, RT‐PCR analysis of left ventricular (LV) GPR120. Each mRNA was corrected for an mRNA level of *cyclophilin*. Results are mean ± SD of three independent experiments. The number of animals in each group is indicated in parentheses. *P < .05 compared with sham; ^†^ *P* < .05 compared with the vehicle‐treated infarcted group

The average weight of rats did not differ among the four groups at baseline (data not shown) and at the end of the study (Table 1). The mortality rate was similar between the two infarcted groups during the experimental period. Relative heart weights corrected for body weight at the end of the study (12 weeks of age) are presented in Table 1. Relative weights of the hearts corrected for body weight at the end of the study (4 weeks after infarction) are presented in Table 1. Four weeks postinfarction, the infarcted areas of the LVs were extensively replaced by scar tissue (Figure 1A). However, in the infarcted groups, the weights of the LVs including the septum were essentially unchanged after 4 weeks. The infarcted rats that received IPE treatment had a significantly lower lung weight/body weight ratio, an index of lung oedema, compared to the infarcted rats treated with vehicle. In addition, the +*dp*/*dt* and −*dp*/*dt* values were significantly higher in the IPE‐treated infarcted group compared to the vehicle‐treated infarcted group. However, there were no significant differences in LV end‐systolic pressure, LV end‐diastolic pressure and infarct size between the infarcted groups.

**TABLE 1:** Cardiac morphology, haemodynamics and biochemistry at the end of study

Parameters	Sham		Infarction	
	Saline	IPE	Vehicle	IPE
No. of rats	10	10	12	12
Mortality, n (%)	0 (0%)	0 (0%)	6 (33%)	5 (29%)
Body weight, g	352 ± 21	378 ± 18	349 ± 16	373 ± 18
Heart rate, bpm	398 ± 20	388 ± 19	403 ± 17	406 ± 17
LVESP, mm Hg	101 ± 5	102 ± 6	105 ± 8	107 ± 7
LVEDP, mm Hg	6 ± 1	5 ± 3	19 ± 4*	17 ± 5*
+*dp*/*dt*, mm Hg/s	6963 ± 364	7102 ± 376	2872 ± 342*	3451 ± 304^*^, ^†^
−*dp*/*dt*, mm Hg/s	6671 ± 337	6592 ± 392	2263 ± 296*	2726 ± 318^*^, ^†^
Infarct size, %	…	…	39 ± 3	40 ± 3
LVW/BW, mg/g	2.06 ± 0.21	2.22 ± 0.25	3.35 ± 0.39*	3.29 ± 0.38*
RVW/BW, mg/g	0.62 ± 0.12	0.65 ± 0.15	0.79 ± 0.11*	0.69 ± 0.10^*^, ^†^
LungW/BW, mg/g	4.22 ± 0.38	4.38 ± 0.29	5.98 ± 0.61*	4.78 ± 0.45^*^, ^†^
Resolvin E1, pg/mL	762 ± 142	19 877 ± 248^‡^	892 ± 172	21 634 ± 435^†^

Note

Values are mean ± SD.

Abbreviations: BW, body weight; LungW, lung weight; LVEDP, left ventricular end‐diastolic pressure; LVESP, left ventricular end‐systolic pressure; LVW, left ventricular weight; RVW, right ventricular weight.

* *P* < .05 compared with respective sham.

^†^ *P* < .05 compared with the infarcted group treated with vehicle.

^‡^ *P* < .05 compared with sham treated with saline.

Besides, the levels of Resolvin E1, a bioactive oxygenated product of EPA, in the IPE‐treated infarcted group was 21 634 ± 435 pg/mL, significantly higher than vehicle (892 ± 172 pg/mL, *P* < .0001), implying the adequate dose of EPA used in this study.

#### 3.1.1 Effect of IPE on GPR120 expression

We investigated GPR120 regulation in the heart of rats. GPR120 protein was significantly increased after MI compared with sham (Figure 1B). However, after administering IPE, GPR120 protein levels were further increased accompanied by a similar change of GPR120 immunohistochemical staining (Figure 1B,C). The changes of *GPR120* mRNA mirrored those in GPR120 protein of Western blot (Figure 1D).

#### 3.1.2 Effect of IPE on oxidative‐nitrosative stress

**FIGURE 2:**
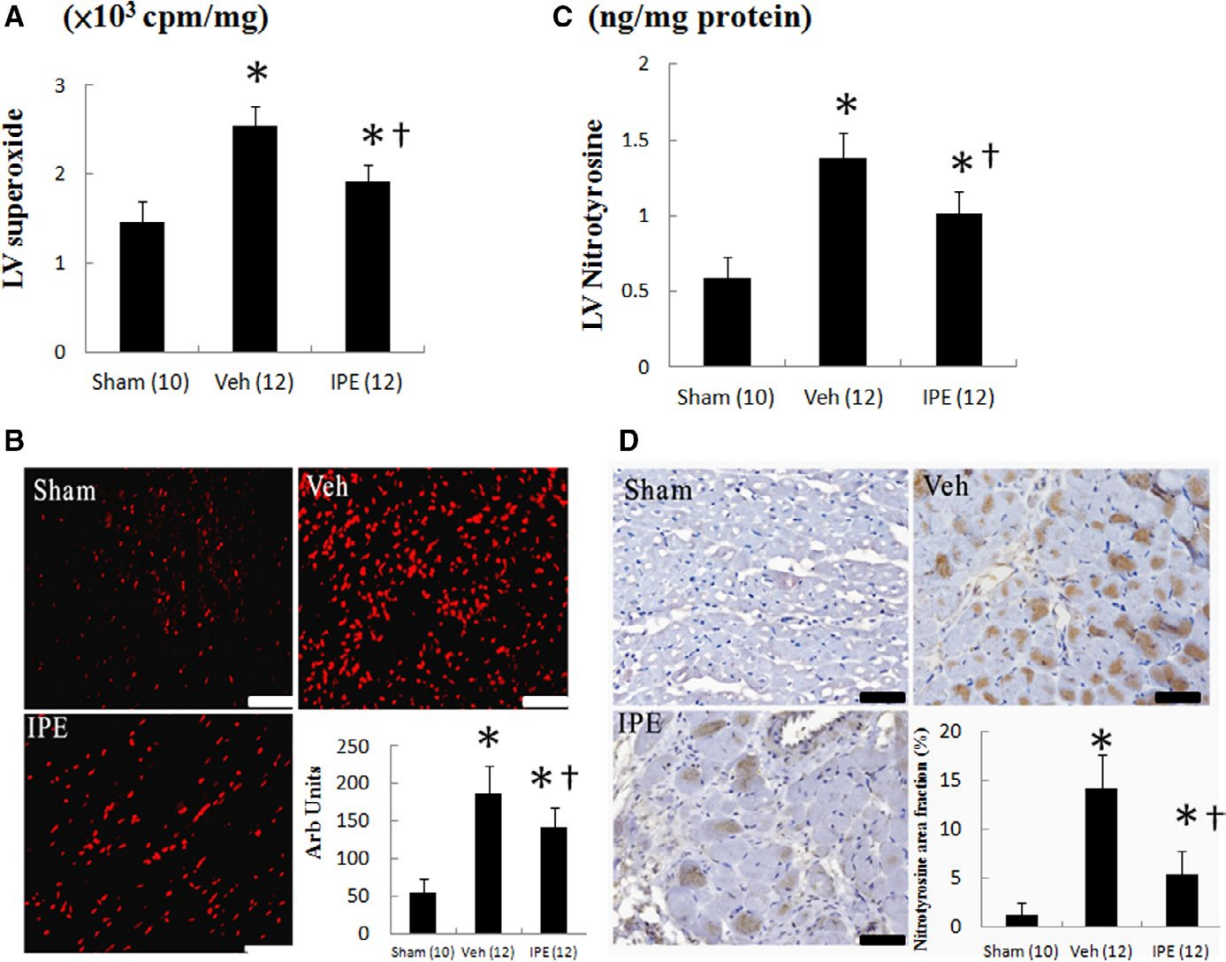
A, Myocardial superoxide by chemiluminescence, B, dihydroethidium (DHE) staining and quantitative analysis, C, nitrotyrosine by ELISA and D, nitrotyrosine immunoreactive staining and quantitative analysis from the remote zone. Myocardial DHE (red fluorescent) and nitrotyrosine (brown) staining showed more intense signals (nuclear position for DHE and cytoplasm for nitrotyrosine) after MI. The number of animals in each group is indicated in parentheses. Bar = 50 μm. *P < .05 compared with sham; ^†^ *P* < .05 compared with the vehicle‐treated infarcted group

Following MI, superoxide production assessed by lucigenin‐enhanced chemiluminescence was markedly increased in the remote LV myocardium as compared with sham (*P* < .001, Figure 2A). Superoxide production significantly fell in IPE‐treated infarcted rats compared with vehicle.

Dihydroethidium reacts with superoxide anions to form a specific nuclear red fluorescence as a marker of superoxide radical generation. As shown in Figure 2B, postinfarction remodelling markedly enhanced the intensity of the DHE staining in the remote zone compared with sham. However, the intensity of the fluorescent signal in the IPE‐treated group was significantly reduced compared with the vehicle‐treated group.

Similarly, LV nitrotyrosine levels in vehicle‐treated infarcted rats significantly increased as compared to sham (*P* < .001, Figure 2C). Myocardial nitrotyrosine levels can be significantly reduced after administering IPE. Nitrotyrosine immunoreactivity, as evidenced by increased brown staining (Figure 2D), was significantly increased after MI, which can be attenuated after adding IPE.

#### 3.1.3 Effect of IPE on cardiac Cx43 phosphorylation

**FIGURE 3:**
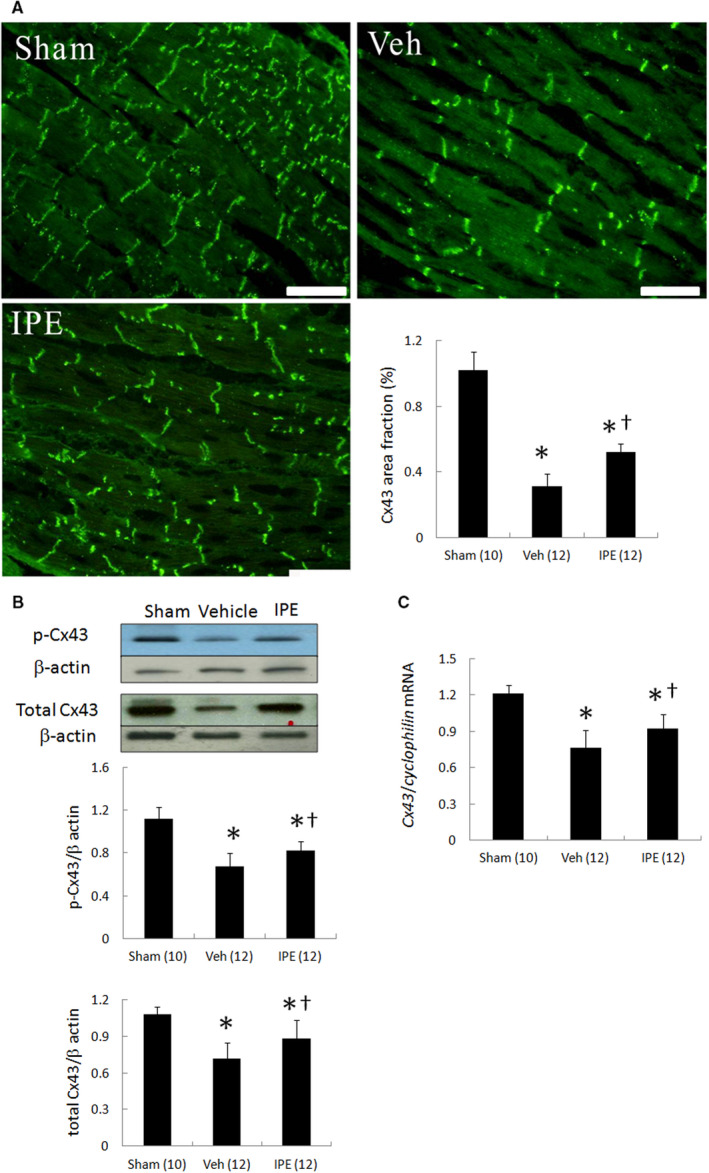
In vivo studies. Immunohistochemistry, Western blot, and RT‐PCR analysis of left ventricular (LV) Cx43 from the remote zone. A, Immunofluorescent staining for Cx43 (magnification 400×). Infarction markedly decreased Cx43 levels. IPE administration partially blocked infarct‐induced Cx43 decrease. Bar = 50 μm. B, Western blot of phosphorylated Cx43 and total Cx43 protein. C, mRNA levels of *Cx43*. Ventricular remodelling after MI was associated with marked decrease in phosphorylated‐ and total Cx43 proteins. Phosphorylated‐ and total Cx43 proteins and *Cx43 mRNA* expression were significantly retained in the IPE‐treated infarcted group compared with the vehicle‐treated infarcted group. Each point is an average of three separate experiments. Relative abundance was obtained by normalizing the density of Cx43 protein against that of β‐actin. Each mRNA was normalized to an mRNA level of *cyclophilin*. The number of animals in each group is indicated in parentheses. *P < .05 compared with sham; ^†^ *P* < .05 compared with the vehicle‐treated infarcted group

To investigate the cardiac Cx43 after infarction, we analysed immunofluorescence, Western blot and mRNA levels. The immunohistochemical analysis showed that in the sham group, sections revealed discontinuous punctate labelling for Cx43 primarily at intercalated discs between two adjacent cardiomyocytes. Infarction resulted in a significant decrease of 70% in the amount of Cx43 immunoreactive signals. The immunoreactive signal of Cx43 was significantly increased in IPE‐treated infarcted rats compared to the vehicle‐treated infarcted rats (Figure 3A).

Western blot showed that ventricular remodelling after MI was associated with significantly decreased levels of p‐Cx43 (ser368) and total Cx43 in the remote zone (Figure 3B). In the IPE‐treated infarcted group, this decrease in level of p‐Cx43 (ser368) and total Cx43 levels was significantly attenuated compared with the vehicle‐treated infarcted group, consistent with the immunohistochemical analysis.

The *Cx43* mRNA level showed significant down‐regulation in the remote zone after MI (Figure 3C). The *Cx43* mRNA level showed a 21% up‐regulation in the remote zone after adding IPE compared with those in the vehicle‐treated infarcted rats (*P* < .001). Thus, the mRNA levels paralleled the changes in the tissue protein levels, implying that the production of *Cx43* mRNA is an essential regulatory step to ensure its local activation.

#### 3.1.4 Effect of IPE on arrhythmias

**FIGURE 4:**
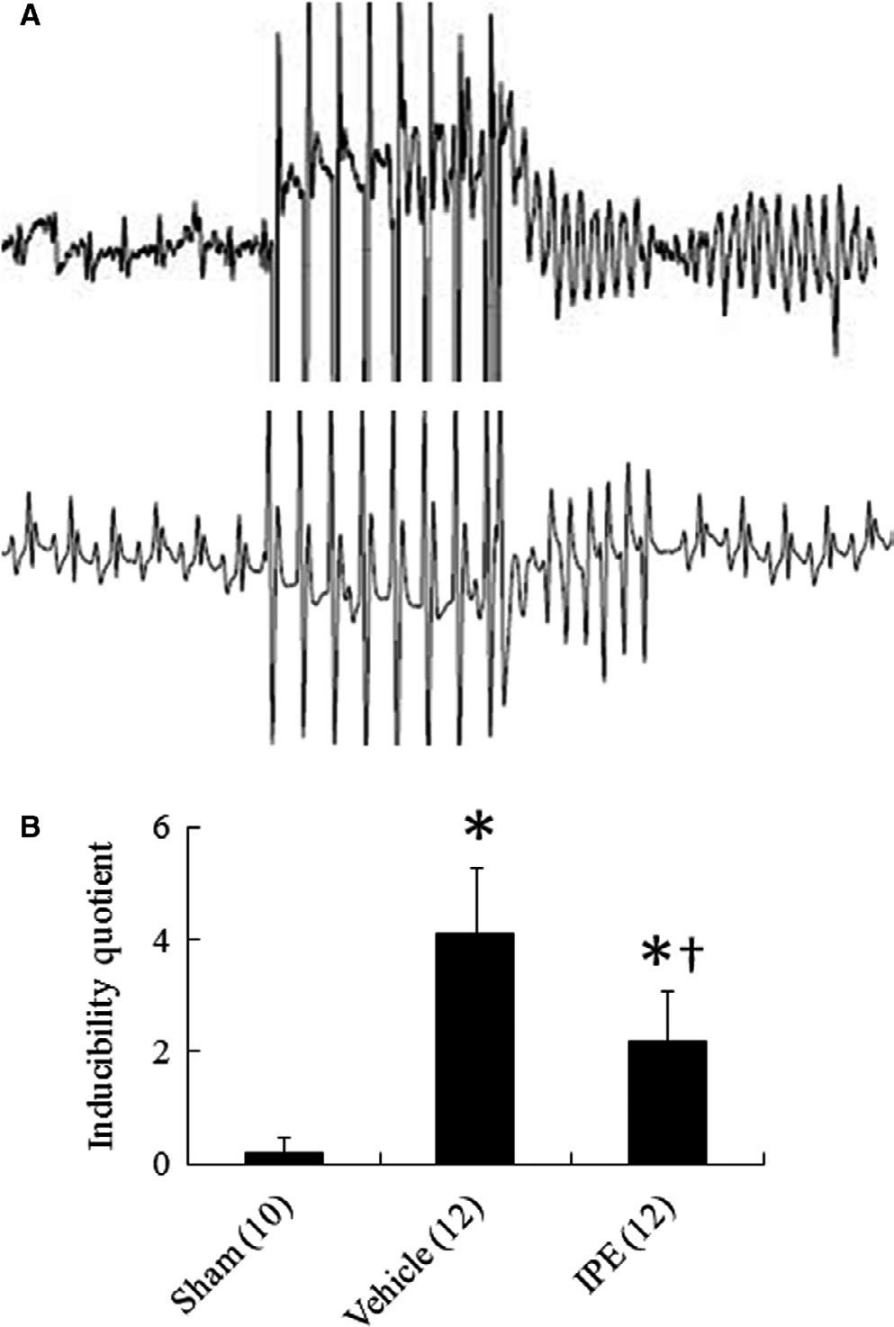
A, representative ventricular arrhythmias induced by ventricular pacing. *Upper panel*: infarcted rat treated with vehicle. Following 8 basic stimuli at a cycle length of 120 ms, sustained tachyarrhythmia was induced (score: 7). *Lower panel*: infarcted rat treated with IPE (score: 5). B, inducibility quotient of ventricular arrhythmias by programmed electrical stimulation 4 wk after MI. The number of animals in each group is indicated in parentheses. *P < .05 compared with sham; ^†^ *P* < .05 compared with the vehicle‐treated infarcted group

To further elucidate the physiological effect of increased Cx43 phosphorylation, ventricular pacing was performed. Sham‐operated rats had low arrhythmia score (0.2 ± 0.3) (Figure 4). In contrast, myocardial infarcted rats had inducible ventricular tachyarrhythmias consisting of ventricular tachycardia and ventricular fibrillation by programmed stimulation. The inducibility of ventricular tachyarrhythmias was significantly decreased by IPE treatment compared to treatment with vehicle.

### 3.2 Part 2: ex vivo

#### 3.2.1 Experiment 2: IPE attenuates ROS and increased Cx43 levels in a GPR120‐dependent pathway

**FIGURE 5:**
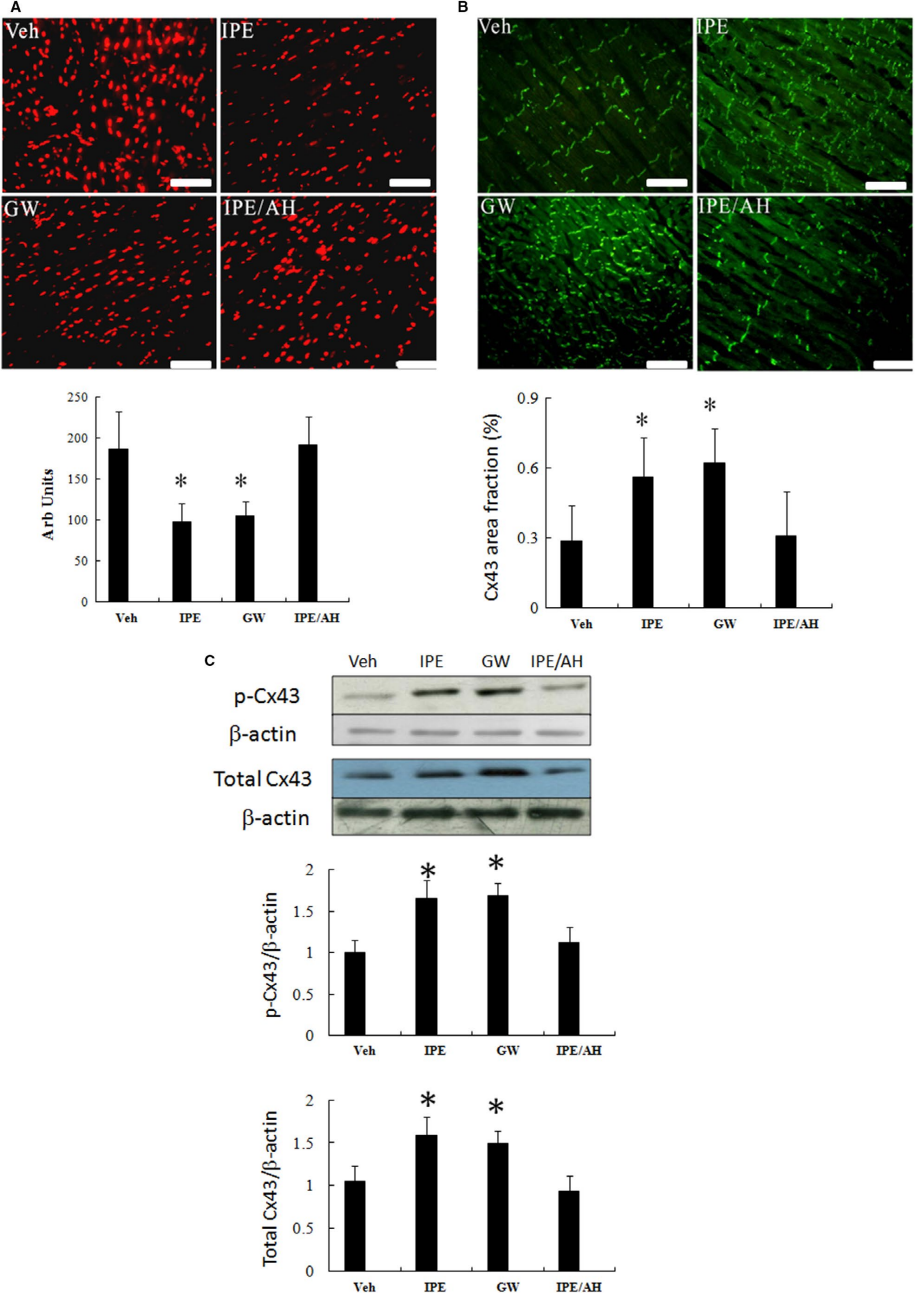
Ex vivo studies. In a rat isolated heart model, the effect of GPR120 on superoxide anions and Cx43 levels is shown. A, dihydroethidium (DHE) staining (magnification 400×) and quantitative analysis. B, Immunofluorescent staining for Cx43 (magnification 400×). C, Western blot of phosphorylated Cx43 and total Cx43 protein. The effects of IPE on superoxide anions and Cx43 levels were reversed by AH‐7614. The effects of GW9508 on superoxide anions and Cx43 levels were similar to those of IPA. Bar = 50 μm. Each point is an average of three separate experiments (n = 5 per group). *P < .05 compared with groups treated with vehicle, and IPE + AH‐7614

The requirement of GPR120 in the regulation of ROS production was shown in infarcted rats from IPE and from the combination of IPE and AH‐7614. As shown in Figure 5A, AH‐7614 reversed the attenuated ROS levels compared with IPE alone, implying that GPR120 is a downstream molecule of IPE.

To further determine whether IPE is incorporated into the membrane phospholipids, we used the GPR120 agonist GW9508. As shown in Figure 5A‐C, when infarcted hearts were treated with GW9508, the extent of antioxidation and increased Cx43 effect was similar to that of IPE. AH‐7614 inhibited the increased Cx43 phosphorylation compared with IPE alone, confirming the critical role of GPR120 in regulation of ROS production and Cx43 levels.

#### 3.2.2 Experiment 3: the role of ROS signalling in IPE‐mediated Cx43 phosphorylation

**FIGURE 6:**
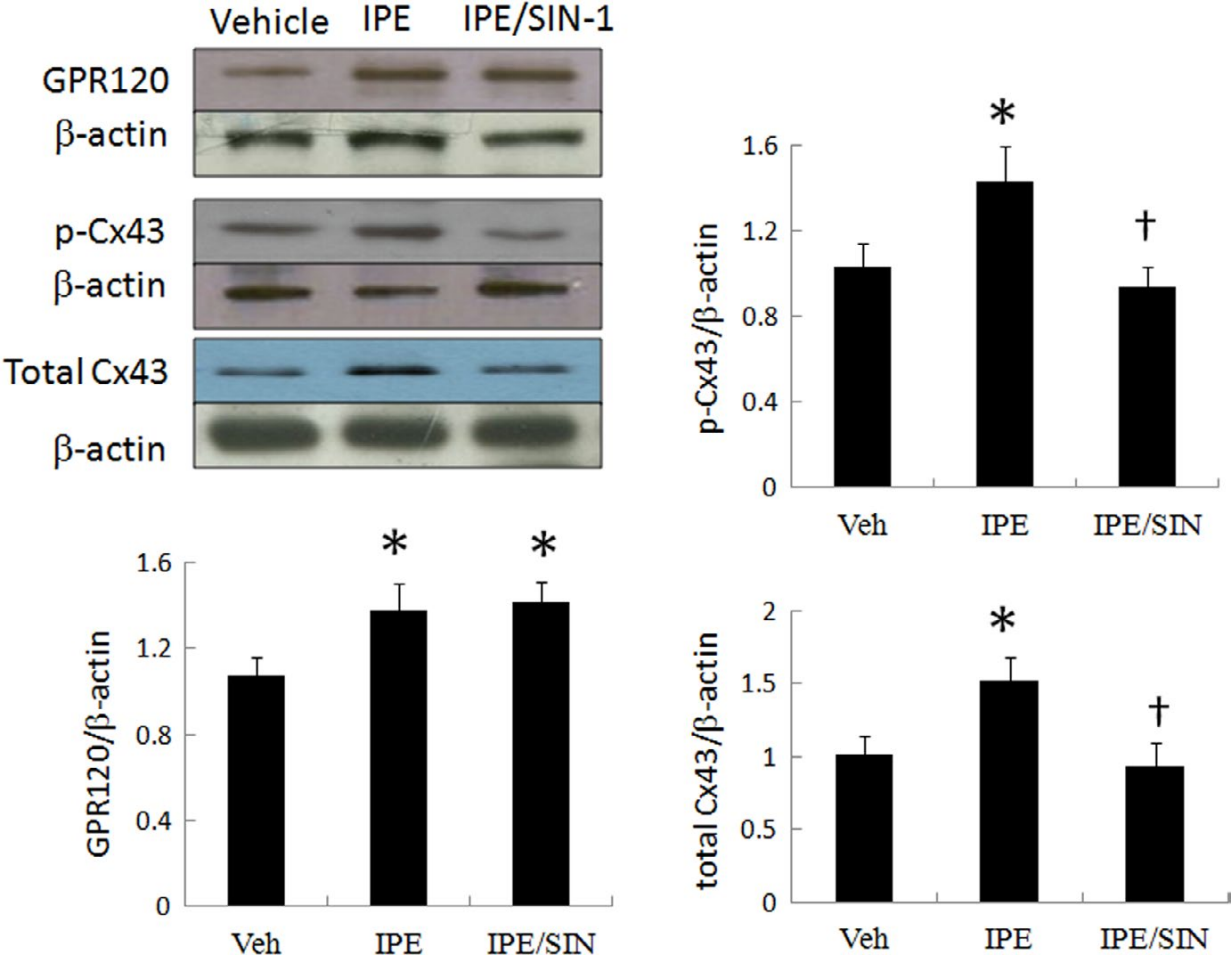
Ex vivo studies. In a rat isolated heart model, the effect of superoxide on IPE‐mediated Cx43 levels. SIN‐1 significantly decreased Cx43 levels compared with IPE alone without affecting the GPR120 levels. Each point is an average of 3 separate experiments (n = 5 per group). *P < .05 compared with vehicle; ^†^ *P* < .05 compared with the IPE‐treated infarcted group

To elucidate the role of the superoxide signalling in IPE‐mediated Cx43 phosphorylation, SIN‐1 was assessed in an ex vivo model. Figure 6 shows that SIN‐1 significantly reversed IPE‐mediated Cx43 phosphorylation compared with IPE alone, whereas GPR120 levels were not affected following the administration of SIN‐1. Thus, ROS were the upstream to modulate the Cx43 phosphorylation.

## 4 DISCUSSIONS

In this study, we explored a novel mechanism by which antiarrhythmias by administering IPE were related to increased Cx43 phosphorylation via GPR120‐mediated ROS production after infarction. To address the role of GPR120 in mediating IPE‐attenuated ROS more directly, we used GPR120 antagonist and agonist to test the requirement for GPR120 to prevent MI‐induced ROS. These results were consistent with the beneficial effects of IPE, as documented structurally by DHE, nitrotyrosine staining and immunofluorescence‐stained Cx43; molecularly by myocardial Cx43 protein and mRNA levels; biochemically by tissue superoxide chemiluminescence, nitrotyrosine and resolving E1 levels; and functionally by improvement of pacing‐induced ventricular tachyarrhythmias. Our study shows that nonpharmacological therapeutics with IPE inhibit postinfarcted arrhythmias in concentrations that are clinically relevant in both in vivo and ex vivo models. Our results were consistent with the notion that a high ω‐3 index is associated with a low‐risk for sudden cardiac death.^30^

**FIGURE 7:**
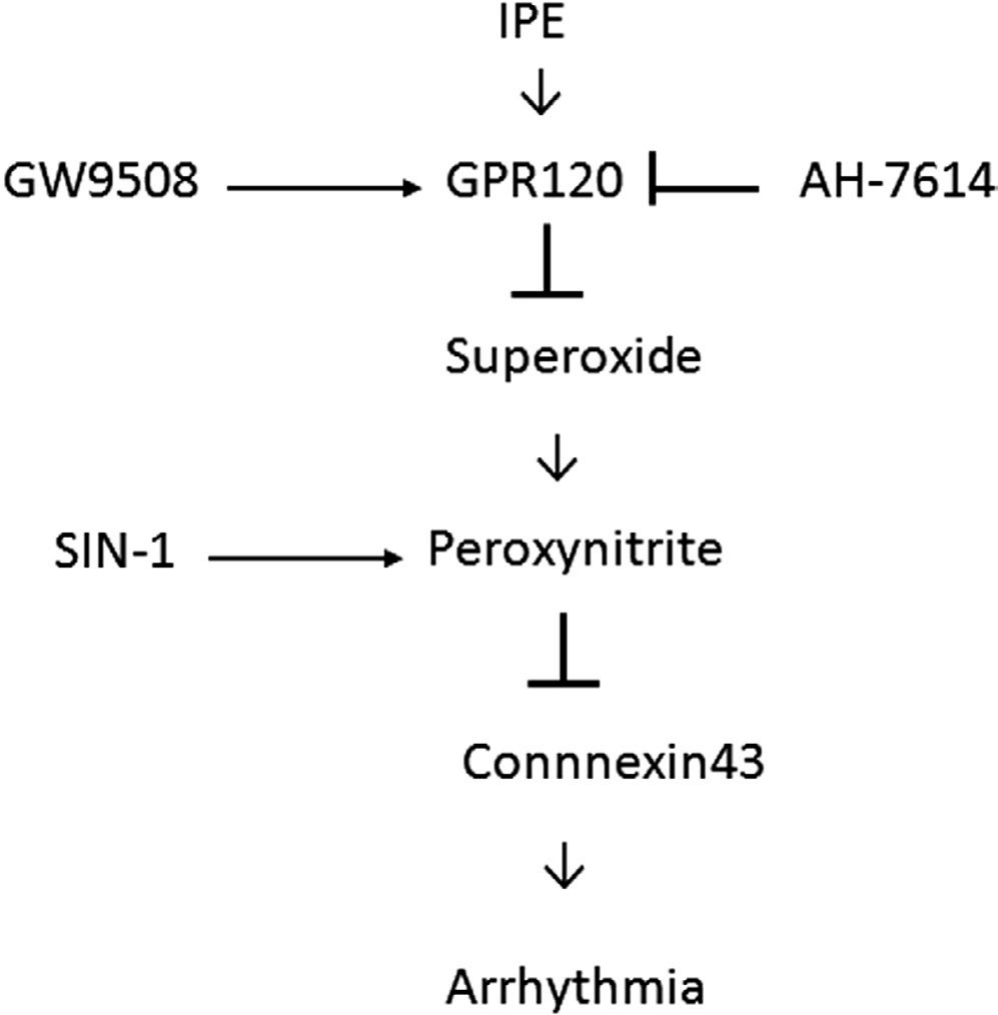
Schematic representation illustrates the involvements of IPE and its related components in Cx43 phosphorylation in postinfarcted rats. IPE inhibits reactive oxygen and nitrogen species through GPR120 activation

Besides nutritional effects, ω‐3 PUFAs are well known to act as intracellular mediators. We showed that increased Cx43 phosphorylation may be an additional mechanism by which chronic treatment for 4 weeks with IPE may be effective therapeutics to improve fatal arrhythmias. Our conclusions are supported by 2 lines of evidence (Figure 7).

1. MI was associated with high oxidative stress, leading to Cx43 loss. The balance between oxidants and antioxidants was disrupted in favour of prooxidants in postinfarcted myocardial remodelling. The role of myocardial superoxide mediating myocardial Cx43 was confirmed by administering SIN‐1. In this study, we demonstrated that SIN‐1 abolished the increased IPE‐mediated Cx43 phosphorylation. Our results confirmed and extended previous findings, showing that disturbances to the nitroso‐redox balance can have a deleterious effect on Cx43 proteins.^15^ Pharmacologic or dietary interventions for correcting this imbalance may have a positive impact on arrhythmias.
2. IPE administration increased the mRNA and protein levels of GPR120. Free fatty acids are generated during lipolysis and enter the bloodstream to circulate throughout the body. Circulating free fatty acids not only provide substrate for energy production but importantly can also act as lipid sensors and mediate the expression of genes and proteins to regulate lipid and energy homeostasis in a diverse range of physiological and pathophysiological conditions. The rats fed with the high‐fat diet significantly up‐regulated the abundance of *GPR120 mRNA* in cardiac tissue,^31^ suggesting that the GRP120 could serve as a dietary fatty acid sensor sensitive to levels of serum fatty acids. Similarly, the *GPR120* mRNA expression was significantly increased after administering DHA determined by RT‐PCR analyses in mouse primary hepatocytes.^32^ Besides, the protein level of GPR120 has been shown to be increased significantly in RAW264.7 cells after EPA treatment,^33^ which was consistent with our findings. Regulatory mechanisms underlying the expression of GPR120 are not understood. Further investigation is required to resolve how IPE regulate the mRNA expression and protein levels of GPR120 in myocardium.
3. IPE as an antioxidant in myocardium was found to be GPR120‐dependent. We found that IPE interacts with GPR120, which in turn attenuated oxidative and nitrative products; correspondingly, GPR120 participates significantly to increased Cx43 phosphorylation. This localization of GPR120 enables the clustering of cell surface receptors with downstream effector molecules. The ability of IPE to inhibit ROS was abolished in GPR120 antagonist. Presence of GPR120 antagonist during IPE exposure resulted in increased ROS production. These findings indicate that the inhibitory effect of IPE on ROS occurs via GPR120. Although previous studies have shown that ω‐3 PUFAs also act through incorporation into cell membranes and modulation of intracellular signalling, the onset of these biological effects may take longer time.^34^ Our experiments with GW9508 showed rapid changes of signalling pathways within 1 hour, implying that activation of GPR120 is sufficient to prevent MI‐induced ROS without affecting membrane lipid composition. Indeed, our results were consistent with the findings of Amos et al,^17^ showing that ω‐3 PUFAs alter the balance of redox homeostasis by activation of the Nrf2 pathway.

As we showed here that postinfarction was associated with increased GPR120 expression, reactive oxygen and nitrogen species (RONS) activity was expected to be reduced if GPR120 was the only upstream to modulate the RONS levels. Indeed, a number of proteins have been reported to associate with RONS and regulate its activity, such as endothelin‐1 and angiotensin II. These RONS‐interacting proteins function to mediate the crosstalk of RONS with other cellular signalling pathways. Attenuated RONS production by increased GPR120 expression after MI alone might not have reached a threshold beyond which benefits occur. Thus, it is not surprising to know that the RONS activity is increased after MI, a status of high GPR120. Thus, increased GPR120 activity by either pharmacology or diet supplement may be as a novel therapeutic target.

### 4.1 Previous studies

The beneficial actions of ω‐3 PUFAs on sudden death have been reported.^1^ However, in some studies ω‐3 PUFA supplementation did not improve the cardiovascular events. The Alpha OMEGA and OMEGA randomized trials included patients who had suffered a MI, and neither showed any improvement in reducing the rate of major cardiovascular events following ω‐3 PUFA supplementation.^35^, ^36^ Similarly, the European Medicines Agency has confirmed that ω‐3 PUFAs containing a combination of ethyl ester of EPA and DHA at a dose of 1 g/d is no longer recommended for secondary prevention of cardiovascular events after MI. The apparent discrepancy among precedent literature may be explained by dose and population, the key crucial factors that act synergistically to determining effective therapy. First, given a threshold dose effect of EPA, the low‐dose supplementation with EPA (226 mg/d, 30; 460 mg/d, 30) cannot provide benefits in the cardiovascular events. Second, previous studies have shown that after a 6‐week dietary intervention with ω‐3 PUFAs PUFAs, ventricular tachycardias were reduced only in subjects with low levels of ω‐3 PUFAs PUFAs.^37^ Thus, use of a low ω‐3 index as an inclusion criterion may allow for more efficient interventional trials, and this may help to elucidate the mechanisms of action. Furthermore, EPA has been shown to enhance mitochondrial function only under conditions where mitochondrial dysfunction is evident. Ventricular remodelling after MI is a process during which mitochondrial dysfunction plays a role.^38^ Indeed, in the current study, we found improvements in reducing ventricular vulnerability in a condition of mitochondrial dysfunction when an adequate dose of EPA (approximately 4 g/d) was given to infarcted rats. The effectiveness of EPA used in this study was confirmed by the results that IPE increased Resolvin E1 levels. Our findings were consistent with previous studies, showing that supplementation studies providing 3.1‐8.4 g EPA + DHA/d have reported 30%‐55% decreases in the production of ROS by stimulated human neutrophils.^39^, ^40^, ^41^ Studies using lower doses of long‐chain ω‐3 PUFAs (0.55‐2.3 g/d) failed to demonstrate effects on ROS production.^42^ Before ruling out the potentially therapeutic beneficial effects of ω‐3 PUFA supplementation, attention must be given to the dose used and what kind of population treated.

### 4.2 Other mechanisms

Although the present study suggests that the mechanisms of IPE‐triggered ROS‐mediated Cx43 expression, other potential mechanisms for antiarrhythmic effect of IPE need to be studied. First, Resolvin E1 possesses anti‐inflammation activities via ChemR23 receptors by inhibiting polymorphonuclear leucocyte transendothelial migration and IL‐12 production.^43^, ^44^ Excessive inflammation has been linked to increased incidence of postinfarction arrhythmia.^45^ Given AH 7614, an GPR120 antagonist, blocked the antioxidation and reversed the increased Cx43 levels of IPE, the possibility of Resolvin E1 in attenuated arrhythmias is unlikely. Second, ω‐3 PUFAs have been shown to inhibit NLRP3 inflammasome activation,^46^ which in turn reduces the downstream molecule of IL‐1β. Given IL‐1β reduced Cx43 protein,^47^ ω‐3 PUFA administration may also preserve Cx43 protein by inhibition of NLRP3 inflammasome‐IL‐1β axis.

### 4.3 Clinical implication

Our results suggest that IPE would have antiarrhythmic effects. The results are complementary to the AHA recommendation that ω‐3 PUFA supplementation is reasonable in patients with prior coronary artery disease.^48^ Traditional antiarrhythmic drug trials have yielded disappointing results, paradoxically precipitated by proarrhythmia. Potential synthetic agonists of GPR120 based on molecular modelling could be further explored to obtain clinical benefits. Our results may open new avenues in our thinking about the action of EPA‐derivatives on critical processes like arrhythmias. Although meta‐analysis showed that ω‐3 PUFA supplements do not improve cardiovascular outcomes in patients with prior coronary heart disease,^21^ a very recent trial using the same agent as this study, Reduction of Cardiovascular Events with Icosapent Ethyl–Intervention Trial (REDUCE‐IT), showed a marked reduction in cardiovascular events.^49^

### 4.4 Study limitations

There are some limitations in the present study that have to be acknowledged. First, given the effect of EPA is both tissue and species specific,^50^ the implications may have limitations when extrapolating data from tissue to tissue or species to species. Second, all of the studies were conducted using pharmacological inhibition, and pharmacological inhibitors may have many potential nonspecific targets. It would be interesting to further evaluate in vivo relationships between the GPR120 pathway and the antiarrhythmic effect of IPE using *GPR120* knockout animal models. However, the existing *GPR120* knockout mouse models exhibit significant hyperglycaemia.^51^ Due to the potential confounding effects of life‐long hyperglycaemia on arrhythmia, we chose to use a pharmacological approach to inhibit GPR120.

## 5 CONCLUSIONS

These data show that IPE at human achievable doses presents beneficial effects on attenuating ROS by activating GPR120, which increased Cx43 phosphorylation after infarction. These effects probably are functionally important because they are linked to attenuated vulnerability of fatal arrhythmias.

## CONFLICT OF INTEREST

The authors confirm that there are no conflicts of interest.

## AUTHOR CONTRIBUTION

**Wei‐Ting Chen:** Conceptualization (supporting); Project administration (supporting); Validation (supporting); Visualization (supporting); Writing‐original draft (lead); Writing‐review & editing (lead). **Syue‐yi Chen:** Data curation (supporting); Formal analysis (supporting); Project administration (lead); Validation (supporting); Visualization (lead); Writing‐original draft (supporting); Writing‐review & editing (supporting). **De‐Wei Wu:** Data curation (supporting); Methodology (supporting); Project administration (supporting); Visualization (supporting); Writing‐original draft (supporting); Writing‐review & editing (supporting). **Cheng‐Che Lee:** Conceptualization (supporting); Formal analysis (supporting); Methodology (supporting); Project administration (supporting); Software (supporting); Validation (supporting); Visualization (supporting); Writing‐original draft (supporting); Writing‐review & editing (supporting). **Tsung‐Ming Lee:** Conceptualization (lead); Data curation (supporting); Formal analysis (lead); Funding acquisition (lead); Investigation (supporting); Methodology (supporting); Project administration (supporting); Resources (lead); Software (lead); Supervision (lead); Validation (lead); Visualization (supporting); Writing‐original draft (supporting); Writing‐review & editing (supporting).

## Supporting information

Appendix S1

## Data Availability

The data that support the findings of this study are available from the corresponding author upon reasonable request.
