# Supplementary material for: Effect of icosapent ethyl on susceptibility to ventricular arrhythmias in postinfarcted rat hearts: Role of GPR120‐mediated connexin43 phosphorylation
Source: J Cell Mol Med. 2020 Jul 8;24(16):9267–79. doi: 10.1111/jcmm.15575 (PMC7417730; doi:10.1111/jcmm.15575)
Supplement: Supplementary file 1 — Appendix S1 [file JCMM-24-9267-s001.docx]

**Supplementary Methods**

***Hemodynamics measurements***

Hemodynamic parameters were measured in anesthetized rats. A polyethylene Millar catheter was inserted into the LV and connected to a transducer (Model SPR-407, Miller Instruments, Houston, TX) to measure LV systolic and diastolic pressure as the mean of measurements of five consecutive pressure cycles as previously described (1). The maximal rate of LV pressure rise (+dP/d*t*) and decrease (-dP/d*t*) was measured.

***In situ detection of superoxide and nitrotyrosine***

For evaluating myocardial intracellular superoxide production using *in situ* DHE (1 µM, Invitrogen Molecular Probes, Eugene, OR, USA) fluorescence, paraffin-embedded tissues (5 µm) were incubated with DHE in PBS (10 mM) in a dark, humidified container at room temperature for 30 minutes. Generation of superoxide radicals by tissue was demonstrated by a red fluorescent signal, and the density of the images was reported as arbitrary units per millimeter square field.

To demonstrate nitrative stress, nitrotyrosine was detected in LV myocardial sections by immunohistochemistry from the remote zone. Nitrotyrosine has been identified as one of the biomarkers for the formation of peroxynitrite, the by-product of ^•^NO and O2 ^•−^. After antigen retrieval and quenching endogenous peroxidase, slides were immunostained with a rabbit anti-nitrotyrosine antibody (1:200, Millipore, Bedford, MA, USA) overnight at 4 °C.

***Real-time RT-PCR of GPR120 and Cx43***

Real-time quantitative reverse transcription-polymerase chain reaction (RT-PCR) was performed from samples obtained from the remote zone (>2 mm outside the infarct) with the TaqMan system (Prism 7700 Sequence Detection System, PE Biosystems) as previously described (3). For rat *GPR120,* the primers were 5'-GACCAGGAAATTCCGATTTG-3' (sense) and 5'-CTGGTGGCTCTCGGAGTATG-3' (antisense). For *Cx43*, the primers were 5′-TACCACGCCACCACCGGCCCA-3′ (sense) and 5′-GGCATTTTGGCTGTCGTCAGGGAA-3′ (antisense). For *cyclophilin*, the primers were 5′-ATGGTCAACCCCACCGTGTTCTTCG-3′ and 5′-CGTGTGAAGTCACCACCCTGACACA-3′. *Cyclophilin* mRNA was chosen as the internal standard because it is expressed at a relatively constant level in virtually all tissues. For quantification, the expression of *GPR120* and *Cx43* was normalized to the expressed housekeeping gene *cyclophilin*. Reaction conditions were programmed on a computer linked to the detector for 40 cycles of the amplification step.

#### *Western Blot Analysis of GPR120, ser368-phosphorylated Cx43, and total Cx43*

Samples obtained from the remote zone at week 4 after infarction. The primary antibodies to GPR120 (Abcam, Cambridge, UK) anti-p-Cx43 (ser368, Chemicon, Temecula, CA, USA; 1:250), anti-total Cx43 (#71-0700; Zymed, San Francisco, CA, USA), and β-actin (Santa Cruz Biotechnology, Santa Cruz, CA) were used. Western blotting procedures were described previously (2). Experiments were replicated three times and results expressed as the mean value.

#### *Immunohistochemical Studies of GPR120 and Cx43*

In order to investigate the spatial distribution and quantification of GPR120 and Cx43, analysis of immunohistochemical staining was performed on LV muscle from the remote zone. Paraffin-embedded tissues were sectioned at a thickness of 5 µm. Tissues were incubated with anti-GPR120 (1:200; Abcam, Cambridge, UK) and anti-Cx43 antibodies (1:200; Zymed, #71-0700) in 0.5% BSA in PBS overnight at 37^0^C. The secondary antibody was monoclonal goat anti-mouse IgG conjugated to fluorescein isothiocyanate (FITC, Sigma), at 1:50 dilution in PBS containing 0.5% BSA for 1 hour. Isotype-identical directly conjugated antibodies served as a negative control.

The slides were coded so that the investigator was blinded to the identification of the rat sections. The density was measured on the tracings by computerized planimetry (Image Pro Plus, Media Cybernetics, Silver Spring, Maryland)) as described previously (2). To eliminate artifactual quantification of Cx43 protein, samples were discarded when the imaging plane was not parallel to the long axis of the fiber. The density was qualitatively estimated from 10 randomly selected fields at a magnification of 400× and expressed as the ratio of labeled nerve fiber area to total area.

**Supplementary references**

1. Lee TM, Lin MS, Chang NC. Effect of ATP-sensitive potassium channel agonists on ventricular remodeling in healed rat infarcts. J Am Coll Cardiol. 2008;51:1309-1318.

2. Lee TM, Chang NC, Lin SZ. [Dapagliflozin, a selective SGLT2 Inhibitor, attenuated cardiac fibrosis by regulating the macrophage polarization via STAT3 signaling in infarcted rat hearts.](https://www.ncbi.nlm.nih.gov/pubmed/28132924) Free Radic Biol Med. 2017;104:298-310.
